# Enhancing Post-Harvest Storability of Kale Using Plasma-Sonic Treatment

**DOI:** 10.3390/foods14234014

**Published:** 2025-11-23

**Authors:** Ji-yeong Jessica Bak, Si-Yeon Kim, Sea C. Min

**Affiliations:** Department of Food Science and Technology, Seoul Women’s University, 621, Hwarangro, Nowon-gu, Seoul 01797, Republic of Korea; jiyeonbak@swu.ac.kr (J.-y.J.B.); asdfisiof123@swu.ac.kr (S.-Y.K.)

**Keywords:** fresh vegetable, microbial decontamination, non-thermal treatment, non-chlorine-based washing

## Abstract

This study investigated a plasma-sonic treatment that combines plasma-activated water (PAW) and ultrasound (US) as an alternative to conventional sodium hypochlorite (NaOCl), which may leave harmful chlorine residues and generate toxic by-products in fresh produce. The treatment was applied to kale to evaluate its decontamination efficiency and storage stability during 7 days at 4 °C. PAW was generated at 52 W and 14.4 kHz for 624 s, and US was applied at 20 kHz and 250 W for 624 s. The plasma-sonic treatment achieved microbial inactivation of indigenous bacteria by 3.2 log CFU/g, which is comparable to the 3.0 log CFU/g reduction achieved by NaOCl treatment. Moreover, the plasma-sonic treatment group exhibited the highest initial moisture content (89.42%) and maintained higher firmness during storage than the NaOCl-washed and untreated groups. Collectively, these findings indicate that the combined PAW and US washing method constitutes a promising non-chlorine-based intervention that enhances microbial stability while maintaining the physicochemical quality of fresh leafy vegetables.

## 1. Introduction

Kale (*Brassica oleracea* L. var. acephala) is a vegetable rich in various bioactive compounds, such as flavonoids, α-tocopherol, β-carotene, and dietary fiber, and is known for its strong antioxidant activity [1]. In general, young leaves with petioles removed from the stem are preferred for consumption in salads and are also commonly used as a source of plant protein, either fresh or in juice [2]. However, kale’s high susceptibility to microbial contamination, coupled with excessive moisture loss during processing and distribution, results in a shortened shelf life, reduced nutrient levels—including soluble antioxidant compounds and chlorophyll pigments—and browning, ultimately diminishing consumer demand [1].

Chlorine-based sanitizers are widely used in the food industry for washing fresh vegetables [3]. However, excessive chlorine concentrations may leave toxic residues on fresh food materials and negatively affect visual quality by causing pigment loss and excessive moisture depletion [4]. Therefore, there is a growing need to develop non-chlorine decontamination technologies that can effectively control viable indigenous microorganisms while maintaining quality characteristics [3].

Plasma-activated water (PAW) is generated through the interaction between plasma discharge and water, either when plasma is produced above the water surface or directly within the liquid [5]. During this process, various reactive species are formed and transferred from the plasma phase to the liquid phase at the gas–liquid interface [5]. These reactive species are mainly classified into reactive oxygen species (ROS) and reactive nitrogen species (RNS) [5]. ROS, including hydroxyl radicals (•OH), singlet oxygen (^1^O_2_), ozone (O_3_), and hydrogen peroxide (H_2_O_2_), are responsible for inducing oxidative stress and membrane damage in microbial cells [5]. RNS, such as nitric oxide (NO), nitrogen dioxide (NO_2_), nitrite (NO_2_^−^), and nitrate (NO_3_^−^), participate in nitration and nitrosation reactions that further contribute to microbial inactivation [5,6]. The concentration and composition of these species depend on the plasma source and operating parameters, which ultimately determine the physicochemical and antimicrobial properties of PAW [6]. The combined presence of long- and short-lived species enables effective microbial decontamination through oxidative and nitrosative mechanisms while minimizing chemical residues [7]. However, in leafy vegetables, such as kale and spinach, irregular surface characteristics and intricately distributed vascular structures may hinder uniform access and adequate contact of plasma reactive species [5]. Consequently, supplementary strategies are necessary to enhance contact efficiency and penetration of plasma reactive species, thereby facilitating the development of an effective PAW-based method for microbial decontamination of leafy vegetables [8].

Ultrasound (US) is a non-thermal decontamination technology that promotes microbial inactivation through cavitation effects [9]. The cavitation phenomenon generates micro-shear forces and micro-jets that physically detach microorganisms from food surfaces, while the radicals and localized physicochemical stresses associated with cavitation induce damage to microbial membranes [8,10]. In addition, it has been reported that the powerful acoustic waves generated during US treatment can enhance the accessibility of PAW to cellular regions that are otherwise inaccessible, thereby amplifying its microbial inactivation efficiency of PAW [11]. Moreover, cavitation-induced effects of US increase the availability of ROS in addition to the reactive species of PAW, resulting in a stronger disinfecting effect of the combined PAW and US treatment [7]. For this reason, the combined application of PAW with US, which may enhance cell membrane permeability and induce localized disruption, facilitates effective penetration of PAW beyond the cell surface, thereby enabling more uniform and effective microbial decontamination in food ingredients [7]. Previous studies on the combined decontamination application of PAW and US have been conducted on chicken [12], crayfish [11], mackerel fillets [7], cherry tomato [9], and celery [13]. However, previous studies have focused on meat and fruit, and relatively little is known about leafy vegetables, such as kale. Moreover, research investigating color, chlorophyll content, and texture as indicators of freshness in leafy vegetables treated with combined PAW and US remains limited. In particular, studies comparing the effect of US treatment using PAW as a treatment medium (“plasma-sonic treatment”) with that of sodium hypochlorite solution washing on the overall physico-chemical properties of kale have not yet been reported. Therefore, the objectives of this study were to (1) evaluate the effect of plasma-sonic treatment on the growth of indigenous bacteria in kale during storage at 4 °C for 7 days, and (2) to investigate the influence of plasma-sonic treatment on the color, chlorophyll content, and moisture loss during storage compared with DW and NaOCl treatments to verify the suitability of non-chlorine decontamination approach for fresh leafy vegetables.

## 2. Materials and Methods

### 2.1. Materials

Fresh kale (*Brassica oleracea* var. acephala) was purchased from a single certified local supplier (Maninsan Agricultural Cooperative Smart APC, Chungnam, Republic of Korea) that was pre-selected before the experiment. The kale was harvested on the same day of purchase and transported to the laboratory under refrigerated conditions (4 ± 1 °C) without any postharvest washing or chemical treatment to ensure sample uniformity and avoid prior sanitization effects.

### 2.2. Sample Preparation

Kale samples were prepared by cutting leaf blades from the petioles. The prepared samples were 12.0 ± 0.5 cm in length and weighed 5.5 ± 0.5 g, respectively. All tools, including scissors, forceps, scalpel, and cutting board, used in preparing the kale samples were sterilized using 70% (*v*/*v*) ethanol before use.

### 2.3. PAW Treatment, US Treatment, Plasma-Sonic Treatment

**Figure 1:**
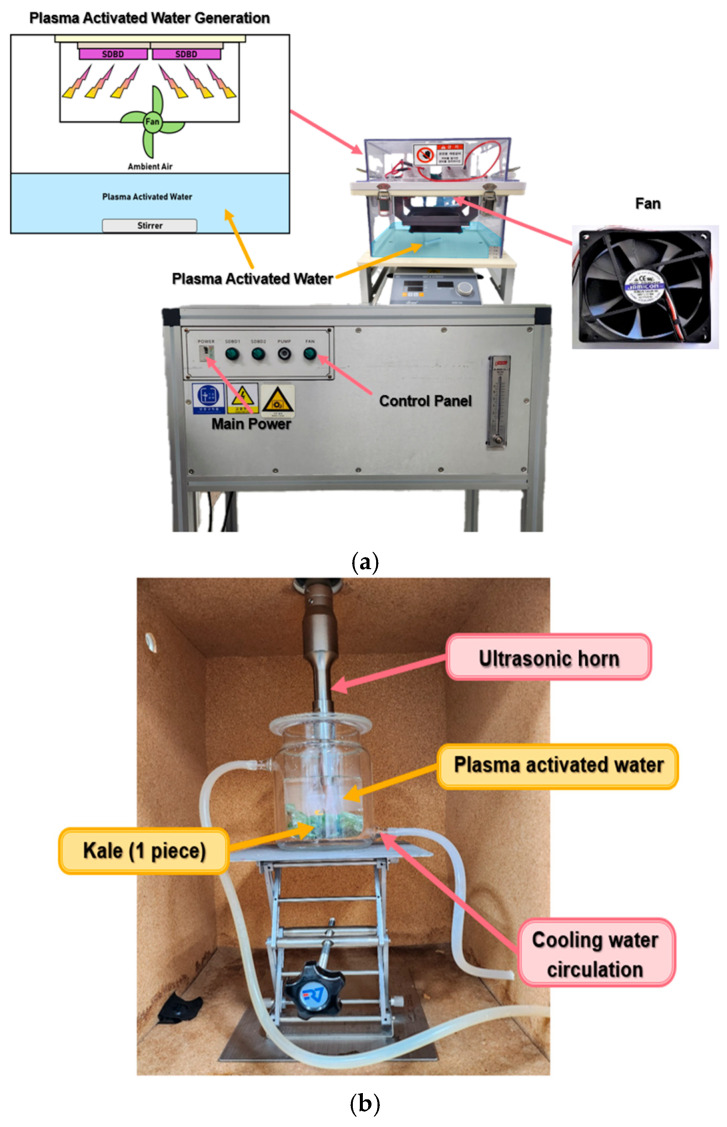
Schematic diagrams of the plasma-activated water (PAW) generation system (**a**) and plasma-sonic treatment system (**b**).

For each treatment, one piece of kale (12 ± 0.5 cm in length, 5.5 ± 0.3 g) was used. The kale sample was immersed in 450 mL of PAW and treated at 150 rpm for 624 s, designated as the “PAW treatment”. PAW was generated in a production chamber (SWU-6; Figure 1a) equipped with two surface dielectric barrier discharge (SDBD) electrodes (8 kV, 52 W, 14.4 kHz). A fan was installed inside the chamber to ensure uniform diffusion of reactive species inside the chamber. Plasma was discharged into 1 L of sterilized distilled water (DW) for 60 min while stirring at 300 rpm and used as the washing solution. The temperature during PAW generation was continuously monitored and maintained below 25–30 °C to ensure nonthermal plasma conditions. Under these conditions, the pH, oxidation–reduction potential (ORP), and electrical conductivity (EC) of the PAW were 2.01 ± 0.01, 597.63 ± 1.09 mV, and 3760 ± 2.09 μS/cm, respectively. These parameters were measured using a pH meter (FiveEasy™ Plus, Mettler Toledo, Greifensee, Switzerland), an ORP meter (Seven Compact S220, Mettler Toledo, Switzerland), and a conductivity meter (K5000-CP, iSTEK, Seoul, Republic of Korea). The obtained values were used to assess the acidity (pH), oxidative capacity (ORP), and ion concentration (EC) of the generated PAW.

A discharge duration of 60 min and a treatment time of 624 s were selected for PAW preparation. The treatment time was determined based on a previous study [14], in which PAW generated under identical discharge conditions exhibited the highest microbial inactivation effect on fresh vegetables. US treatment for 624 s, identical to the PAW treatment time, was designated as the “US treatment”. The US system consisted of a horn-type ultrasonic processor (ULH-700N, Ulsso Hitech, Cheongju, Republic of Korea) operating at 20 kHz and 250 W (Figure 1b). To prevent excessive heating of the kale and water during US treatment, cooling water (15 °C) was circulated through a cooling jacket surrounding the treatment chamber.

Plasma-sonic treatment was defined as US treatment at 20 kHz and 250 W for 624 s, utilizing PAW prepared for 60 min as the treatment medium. During plasma-sonic treatment, cooling water (15 °C) was circulated through a cooling jacket to prevent excessive heating of the kale and water. Other washing methods were designated as follows: washing with sterile distilled water (DW; 450 mL) was termed the ‘DW treatment,’ washing with sodium hypochlorite solution (NaOCl; 100 ppm; 450 mL) was designated the ‘NaOCl treatment,’ and no washing was labeled ‘untreated (NT)’. The NaOCl solution had a pH of 8.7, which is within the recommended range for food sanitation.

### 2.4. Microbial Analysis

To evaluate the antimicrobial effects of each treatment, unwashed kale samples were stored at 36 ± 0.1 °C for 24 h to promote the growth of indigenous mesophilic aerobic bacteria. For the storage experiment, freshly prepared kale samples without prior incubation were used to monitor changes in the microbial population during storage. Subsequently, the samples were subjected to six different treatments: NT, DW, NaOCl, PAW, plasma-sonic, and US. For microbial analysis, each treated kale leaf (10 g) was collected from the same anatomical region of the leaf (middle lamina between the midrib and the margin) to ensure sampling consistency. The samples were then placed in a sterile nylon/low-density polyethylene (Ny-lon/LDPE) bag (Dasanpack, Seoul, Republic of Korea) and homogenized with 90 mL of sterile 0.1% (*w*/*w*) Bacto™ peptone solution (Gibco, Grand Island, NY, USA) at a 1:9 (sample:solution, *w*/*v*) ratio using a stomacher blender (LS-400A; BNF Korea, Gimpo, Republic of Korea) for 5 min. The homogenates were then serially diluted tenfold with sterile 0.1% peptone water (Difco™, Becton Dickinson, Detroit, MI, USA), and aliquots were spread on plate count agar (PCA; Becton Dickinson Company, Franklin Lakes, NJ, USA). PCA plates were incubated at 36 °C for 48 h, and the resulting colony-forming units (CFU) were expressed as the indigenous bacterial count of kale.

### 2.5. Moisture Content, pH, and Color

For physicochemical analyses (moisture content, pH, color, and chlorophyll), kale leaf blades were cut into 0.5 × 0.5 cm^2^ pieces before treatment. To ensure consistency, all samples were collected from the same anatomical region of the leaf (middle lamina between the midrib and the margin). The wet weight of each sample was recorded prior to drying, and the dry weight was measured after drying at 105 °C until a constant weight was achieved (approximately 2 h) using a moisture analyzer (i-Thermo 163 L, Bel Engineering Inc., Milano, Italy). The moisture content was automatically calculated by the analyzer as the percentage of water loss relative to the initial wet weight. The dry residue was determined as the ratio of the remaining dry weight to the initial wet weight, and the A value (%), defined as the ratio between the initial and final sample weights, was obtained directly from the analyzer output. For pH determination, 2 g of kale samples were homogenized with 18 mL of distilled water using a homogenizer (HG-15D, Daihan Scientific, Wonju, Republic of Korea), and the supernatant was analyzed with a pH meter (SFM-T455CP; Shinil, Cheonan, Republic of Korea). Color analysis was conducted on intact kale leaves placed flat on a white background using a colorimeter (CR-400, Konica Minolta Inc., Tokyo, Japan) calibrated with a Minolta standard plate (Y = 87.5, x = 0.3174, y = 0.3352). Chromaticity was measured under Illuminant D65 and expressed in the CIE L*, a*, and b* color space. During the storage test (0, 1, 3, and 7 days at 4 °C), color changes were monitored for NT, DW, NaOCl, PAW, US, and Plasma-sonic treatment. Samples were stored in an acrylic chamber containing a nylon linear low-density polyethylene (Nylon-LLDPE) bag, where temperature (4.38 ± 0.48 °C) and relative humidity (95.00 ± 0.00%) were continuously monitored using a data logger (Testo SE & Co. KGaA, Titisee-Neustadt, Germany).

### 2.6. Texture

The firmness (burst strength) of kale leaves was determined using a texture analyzer (TA/XT2/25, Stable Micro Systems Co., Ltd., Surrey, UK) equipped with a 2 mm diameter stainless-steel cylindrical probe. The firmness of kale was defined as the maximum force (N) required to penetrate the leaf surface, representing the rupture strength of the tissue. The measurement procedure was based on the method of [15] with slight modification. Five kale leaves were stacked, and five random positions per leaf were tested. The pre-test, test, and post-test speeds were each set to 1.0 mm/s, and the strain was adjusted to 40%. Firmness changes during storage (0, 1, 3, and 7 days at 4 °C) were evaluated for all treatment groups (NT, NaOCl, and Plasma-sonic treatment) using the same measurement conditions.

### 2.7. Chlorophyll Content

Chlorophyll content was measured according to the method of [16] with slight modifications. Kale leaf blades were cut into 0.5 × 0.5 cm^2^ pieces, and 1 g of each sample was taken for analysis. The 9 mL of an ethanol solution (94.5%, Daejung Chemicals & Metals, Siheung, Republic of Korea) was added to the sample, followed by sonication at 400 W for 10 min (Powersonic 610; Hwashin Tech, Daegu, Republic of Korea). Centrifugation (12,300× *g*, 5 min; Gyrozen, Gimpo, Republic of Korea) was performed to separate the dye solution from the resist. The absorbance of the dye solution was measured at 663 nm and 645 nm using a UV-vis spectrophotometer (Spectramax-M3, Molecular Devices, San Jose, CA, USA). the contents of chlorophyll a and b were determined using Equations (1) and (2).

(1) $$Chlorophyll\;a\;\left(\frac{mg}{g}\right)=\left(12.7\times A663\right)-\left(2.59\times A645\right)$$

(2) $$Chlorophyll\;b\;\left(\frac{mg}{g}\right)=\left(22.9\times A645\right)-\left(4.7\times A663\right)$$

### 2.8. Statistical Analysis

All experiments were conducted in triplicate as independent biological replicates prepared and treated on separate experimental days. For microbial analysis, two kale samples (12.0 ± 0.5 cm each) were used per treatment within each biological replicate, representing technical duplicates. For color, texture, moisture, and chlorophyll analyses, three analytical subsamples (2 g each, except 1 g for chlorophyll) were used per treatment as technical triplicates. Data were analyzed using one-way analysis of variance (ANOVA) with Tukey’s honestly significant difference (HSD) test (*p* < 0.05) in SPSS Statistics 26 (IBM Corp., Armonk, NY, USA).

## 3. Results and Discussion

### 3.1. Microbial Analysis of Indigenous Bacteria in Kale During Storage

Table 1 presents the effects of various washing treatments on the inactivation of indigenous bacteria on kale. Plasma-sonic treatment exhibited the highest reduction in microbial load, decreasing the initial count (7.2 ± 0.1 log CFU/g) to 3.2 ± 0.1 log CFU/g, which was comparable to the effect of NaOCl treatment (3.0 ± 0.1 log CFU/g). Although US (2.3 ± 0.0 log CFU/g) and PAW (2.0 ± 0.0 log CFU/g) treatments produced higher reductions than DW treatment (1.8± 0.1 log CFU/g), they were less effective than plasma-sonic treatment. Viable bacterial counts in rinsed water showed a similar trend, with NaOCl and plasma-sonic treatments exhibiting the lowest values (3.0 ± 0.3 and 3.6 ± 0.2 log CFU/g, respectively), confirming the effective inactivation of microorganisms detached from the kale surface during washing.

**Figure 2:**
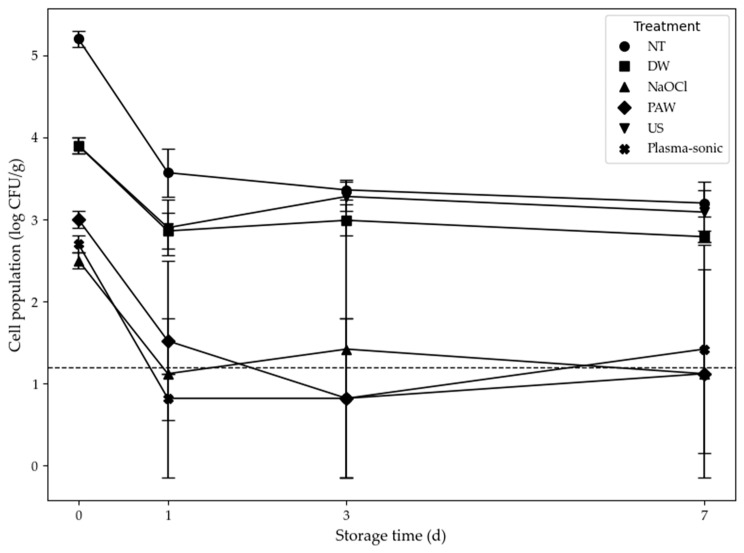
Changes in indigenous bacterial count of kale subjected to various treatment (NT, DW, NT NaOCl, PAW, US, Plasma-sonic). All samples were stored at 4.4 ± 0.5 °C and 95.0 ± 0.1% RH for 7 days during storage. NT: unwashed kale; DW: kale washed using DW; NaOCl: kale washed using NaOCl; PAW: kale washed using PAW; US: kale washed using ultrasound treatment; Plasma-sonic: kale washed using plasma-sonic. The detection limit (1.2 log CFU/g) is indicated by a dotted line.

During storage, plasma-sonic and PAW treatments maintained microbial levels comparable to those of NaOCl-treated samples, indicating equivalent antimicrobial and preservation effects (Figure 2). The strong microbial reduction and stability observed in the plasma-sonic treatment are likely attributed to the PAW component, which exhibited antimicrobial efficacy comparable to that of both NaOCl and plasma-sonic treatments. The microbial count declined from 2.7 ± 0.1 log CFU/g at day 0 to 0.82 ± 0.97 log CFU/g at day 1 and day 3 and remained low at 1.42 ± 1.27 log CFU/g by day 7, closely similar to the level observed in the NaOCl-treated sample (1.12 ± 0.00 log CFU/g). In contrast, the PAW-treated sample initially showed a reduction comparable to NaOCl on day 1, but its count increased thereafter, resulting in higher levels than both plasma-sonic and NaOCl by day 7. The US-treated sample exhibited moderate reductions, with microbial counts at day 7 (3.09 ± 0.37 log CFU/g), still higher than those of plasma-sonic and NaOCl treatments, yet lower than those of DW and PAW.

Fish fillets treated with a combined treatment of PAW (220 V, 800 W, 10 mm, 20 kHz, 10 min) and US (220 V, 4 L, 40 kHz, 100 W ultrasonic input, 500 W total input, 30 min) and stored at 4 °C showed lower total viable counts on days 2–3 (5.76 ± 0.25 log CFU/g) compared with the untreated group (6.55 ± 0.07 log CFU/g) [17]. This reduction in microbial load indicates that the growth of indigenous bacteria was delayed, which consequently delayed spoilage compared with the untreated group [17]. Also, celery treated with PAW (8 kV, 52 W, 14.4 Hz, 338 s) and US (20 kHz, 700 W), either individually or in combination, and then stored at 4 °C, showed on day 0 an inhibition of 3.0 ± 0.3 log CFU/g for the combined treatment, which was higher than that of the NaOCl control (2.6 ± 0.3 log CFU/g), while the individual PAW (2.3 ± 0.3 log CFU/g) and individual US (1.3 ± 0.3 log CFU/g) treatments were lower than the combined treatment and also lower than the untreated groups [14]. These results suggest that the enhanced antimicrobial efficacy of the combined treatment can be attributed to the individual effects of PAW and US.

In particular, PAW generated at 9 kHz and 5 kV on rocket leaves reduced indigenous bacteria by more than 2 log CFU/g over time and showed effects comparable to NaOCl washing at 100 ppm [18]. These antibacterial effects are attributed to reactive species in PAW that damage bacterial membranes, induce leakage of intracellular components, and reduce cell viability [18]. Specifically, ref. [19] reported that PAW generated using a SDBD plasma system operated at 7.75 W and 500 Hz for up to 5 min exhibited pronounced physicochemical changes. Under these conditions, highly RONS including NO•, HO•, NO_2_•, HOO•, and ONOO^−^ were transferred into water and subsequently converted into more stable and biologically active forms such as H_2_O_2_, O_3_, HNO_2_, and HNO_3_ [20]. The concentrations of NO_3_^−^, NO_2_^−^, and H_2_O_2_ increased with treatment time, reaching approximately 292.4, 17.5, and 23.1 mg L^−1^, respectively, after 5 min of plasma exposure. The corresponding pH and ORP of the PAW ranged from 4.1 to 2.6 and 487 to 660 mV, respectively. In the present study, the pH, ORP, and EC of PAW were 2.01 ± 0.01, 597.63 ± 1.09 mV, and 3760 ± 2.09 μS/cm, respectively, showing physicochemical characteristics comparable to those previously reported by [20].

**Figure 3:**
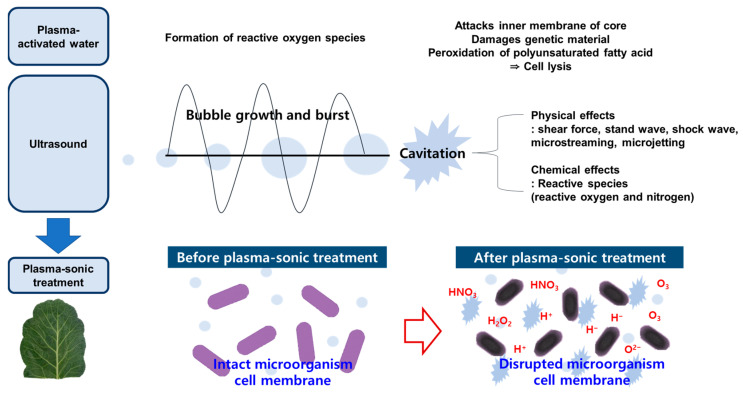
Schematic illustration of plasma-sonic treatment and its antibacterial mechanism.

In addition, US treatment of saffron corms (37 kHz, 100% amplitude) for 60 min/day over 7 days significantly reduced surface microbial contamination compared with the control (*p* < 0.05), demonstrating the antimicrobial potential of US in lowering microbial load [19]. Beyond its direct effect, US generates additional reactive species that act synergistically with the RONS in PAW to promote lipid peroxidation, alter membrane permeability, and cause depolarization, thereby contributing to microbial inactivation [21]. Furthermore, US enhances cell-membrane permeability, which facilitates the penetration of PAW and renders microorganisms more vulnerable to oxidative disruption [21]. Acoustic cavitation also produces physical damage: when cavitation bubbles collapse, microstreaming and shock waves generate micropores and partially rupture cell walls and cytoplasmic membranes [22]. Taken together, the higher inhibitory effect of plasma-sonic treatment on indigenous bacteria in kale can be explained by a dual mechanism in which the micropores and partial structural disruption generated by US accelerate PAW penetration into tissues (Figure 3).

Consequently, microbial counts during storage remained lower than those observed with the individual treatments of DW, PAW, or US. Therefore, plasma-sonic treatment can be considered a practical alternative to chlorine-based washing for applications at 4 °C that require both strong initial inactivation and sustained suppression of indigenous bacterial growth.

### 3.2. Color of Kale After Treatment

**Figure 4:**
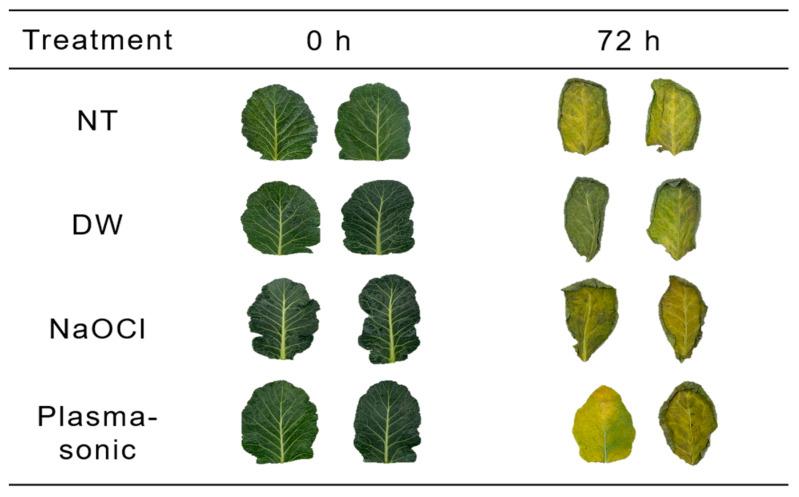
Appearance of kale after various treatment (NaOCl, Plasma-sonic). NT: unwashed kale; NaOCl: kale washed using NaOCl; Plasma-sonic: kale washed using plasma-sonic. All samples were stored at 4.4 ± 0.5 °C and 95.0 ± 0.1% RH for 3 days during storage.

The color parameters and greenness index of kale are presented in Table 2 and the appearance are shown in Figure 4. The L* value was maintained in the NT (38.52 ± 1.26) and plasma-sonic (38.16 ± 0.30) groups, whereas DW (36.38 ± 0.11) and NaOCl (35.78 ± 0.92) treatments showed significant decreases (*p* < 0.05). The a* value was lowest in the plasma-sonic group (−12.24 ± 0.24), followed by the US (−11.07 ± 0.48) and PAW (−8.72 ± 0.19) groups, indicating reduced redness compared with the other treatments, while the NaOCl group showed the highest value (−6.86 ± 0.07). The b* value of plasma-sonic–treated kale (13.69 ± 0.51) was comparable to that of NT (13.02 ± 1.89) and US (13.35 ± 0.92), whereas DW (7.49 ± 1.05) and NaOCl (7.44 ± 0.65) treatments resulted in significantly lower yellowness (*p* < 0.05). Consistently, Δ*E* values were lowest in NT, US, and plasma-sonic groups, while DW and NaOCl showed significantly higher color differences (*p* < 0.05). For the greenness index, plasma-sonic treatment showed the highest OD_665_ and OD_649_ values (0.74 ± 0.01 and 0.43 ± 0.01, respectively), followed by the US group (0.61 ± 0.01 and 0.33 ± 0.01). These groups also exhibited the highest chlorophyll a (8.28 ± 0.13 mg/g for plasma-sonic and 6.89 ± 0.11 mg/g for US) and chlorophyll b (6.37 ± 0.12 mg/g for plasma-sonic and 4.69 ± 0.10 mg/g for US) contents. In contrast, DW- and NaOCl-treated kale showed notably reduced chlorophyll a (4.74 ± 0.09 and 4.43 ± 0.10 mg/g, respectively) and chlorophyll b (3.29 ± 0.08 and 2.75 ± 0.11 mg/g, respectively) contents. Compared with the DW-treated kale, the chlorophyll a and b contents of the plasma-sonic–treated samples were approximately 75% and 94% higher, respectively. Furthermore, when compared with NaOCl-washed kale, chlorophyll a and b contents were 87% and 132% higher, respectively. The superior color retention and higher chlorophyll content observed in the plasma-sonic group can be explained by the reactive species generated in PAW, which inactivated chlorophyll-degrading enzymes [18], and by the structural modifications induced by US [23]. Previous studies have reported immediate decreases in color values after PAW or plasma treatments due to oxidative stress, although long-term stability was attributed to the inactivation of chlorophyll-degrading enzymes [18]. For instance, PAW-treated rocket leaves initially showed reductions in L*, a*, and b* values due to partial chlorophyll degradation; however, further color loss was suppressed during storage as a result of enzyme inactivation [18]. Similarly, plasma-treated kiwi showed an initial reduction in chlorophyll b but exhibited improved color retention during storage owing to the inactivation of color-degrading enzymes [24]. In the case of US, lettuce treated at 34 kHz and 81 W/L showed an increase in L* values during storage, which was associated with dehydration and oxidative processes; however, a*, b*, and Δ*E* values remained nearly unchanged compared with NT, indicating that overall color retention was maintained [23]. Unlike previous research in which PAW or US induced initial pigment loss or color changes, plasma-sonic treatment in this study did not cause excessive chlorophyll oxidation or browning, highlighting its potential advantage in preserving color quality. These results suggest that the preservation of color quality under plasma-sonic treatment is attributable to the suppression of pigment degradation and the minimization of tissue microstructure alterations. Therefore, plasma-sonic treatment can be considered an effective and promising washing method for preserving the appearance and color stability of fresh vegetables during storage.

### 3.3. Quality Attributes of Kale After Treatment and During Storage

The moisture properties and storage quality of kale immediately after NaOCl and plasma-sonic treatments are presented in Table 3 and Table 4. The plasma-sonic group exhibited the highest moisture content (89.42%, wet basis) and water-holding capacity (final/initial weight ratio of 945.93%), demonstrating superior moisture retention compared with the other groups. The kale was considered turgid and appeared hydrated after plasma-sonic treatment (Figure 4). This effect can be attributed to acoustic cavitation generated by US, which induces transient micropores in cell membranes, allowing the washing solution to penetrate into intercellular spaces and form hydrogen bonds with cell wall polysaccharides and proteins, thereby converting free water into bound water [25,26]. This water conversion minimizes evaporation and leaching during storage [26]. Simultaneously, PAW increases cell membrane permeability through RONS, which partially oxidize cell wall components and facilitate deeper water diffusion into the tissue matrix [20]. In this study, the enhanced permeability induced by PAW, together with the mechanical cavitation of US, creates a synergistic effect that promotes water retention, minimizes evaporation and leaching during storage, and maintains tissue firmness and hydration.

The high water-holding capacity was attributed to the maintenance of turgor pressure, which delayed firmness loss during storage [27]. Although firmness decreased in all groups, the plasma-sonic group retained relatively higher firmness than NT and NaOCl (Table 4). Consistent with previous studies, US treatment has been shown to contribute to firmness retention in fresh produce [25]. For instance, in strawberries, US treatment at 20 kHz for 10 min resulted in higher firmness compared with the untreated control during cold storage [27]. Similarly, mushrooms subjected to US at 30 W for 3 min exhibited significantly higher water-holding capacity and firmness, whereas treatments longer than 10 min caused excessive cell damage and reduced firmness [25]. Apples treated with US at 20 kHz for 10 min not only maintained firmness but also showed a 19.4% increase compared with the untreated group after 60 days of storage [28]. Furthermore, PAW treatment has been shown to delay tissue softening and maintain firmness in various fresh produce, including blueberries, mushrooms, and apples, by preserving membrane integrity and reducing electrolyte leakage during storage [29]. Therefore, based on the previous results, it can be suggested that plasma-sonic treatment delayed tissue softening by suppressing moisture loss, primarily through the maintenance of turgor pressure and the reduction in structural damage [25]. This mechanism, in line with the firmness-preserving effects of US reported in strawberries, apples, and mushrooms, underscores the potential of plasma-sonic treatment as a promising non-chlorine-based washing technology for preserving texture quality in fresh vegetables during storage.

The plasma-sonic group also maintained L* and b* values comparable to those of NT and exhibited lower Δ*E* values, which indicated higher color stability. The slight variation observed in NT could be attributed to minor chlorophyll degradation, moisture loss, and surface microstructural changes that naturally occur in leafy vegetables during refrigerated storage. In contrast, DW- and NaOCl-treated samples showed significant decreases in L* and b* values and increased Δ*E* during storage, suggesting reduced color stability. These differences were closely associated with changes in pH [30]. In general, higher pH enhances polyphenol oxidase (PPO) activity, thereby promoting polyphenol oxidation and accelerating browning [30]. This is typically manifested as increased a*, decreased b*, and elevated Δ*E* values [30]. Conversely, lower pH suppresses PPO activity, thereby delaying browning and preserving color stability [31]. Additionally, the higher color change in the DW group may have resulted from increased surface hydration and mechanical stress during washing, which facilitated partial chlorophyll leaching during storage [30]. In this study, the NaOCl group showed a gradual increase in pH during storage, which coincided with reduced color stability, whereas the plasma-sonic group maintained relatively lower and more stable pH values, effectively suppressing PPO activity and delaying browning. Supporting this mechanism, previous studies have reported that fresh-cut potatoes treated with PAW at 200 Hz and 70 kV for 3 min showed significant reductions in PPO and peroxidase (POD) activities [30]. This effect contributed to maintaining lower browning index during storage, as the acidic environment generated by PAW lowered pH and suppressed the activity of browning-related enzymes [30]. Similarly, another study found that fresh produce treated with PAW at 10 kV for 60 s exhibited immediate reductions in PPO activity, which delayed browning and maintain higher lightness values compared with the untreated control [32]. These findings suggest that the color-preserving effect of PAW is primarily attributed to the acidic environment it generates, which lowers pH and thereby inhibits PPO activity [30]. In addition to this pH-dependent mechanism, another important effect involves ROS and RNS produced in PAW, which can directly inactivate PPO by oxidizing its copper center or modifying amino acid residues [33]. Although PPO activity was not directly determined in the present study, the stable L* and b* values and lower Δ*E* observed in the plasma-sonic group are consistent with a mechanism in which both the acidic environment created by PAW and the action of ROS and RNS contribute to the inhibition of PPO activity, thereby delaying enzymatic browning. Also, in the case of US, PPO inactivation occurs through cavitation, where bubble collapse generates localized high temperature, pressure, and shear forces that unfold enzyme secondary structures, while hydroxyl radicals (·OH) produced during cavitation oxidize amino acid side chains and further reduce enzyme activity [34,35]. A previous study reported that sweet potato slices treated with US at 40 kHz for 10 min and stored at 4 °C exhibited inhibited PPO and POD activities, which contributed to improved color stability during storage [36,37]. Although PPO activity was not directly determined in this study, taken together, the superior color stability observed in the plasma-sonic group can be explained by the combined effects of enzyme inactivation and cavitation-induced structural modifications. PAW-derived ROS and RNS suppressed chlorophyll-degrading and browning-related enzymes, while US-induced cavitation created micropores that enhanced PAW penetration without causing excessive tissue damage.

Consequently, the plasma-sonic group maintained high moisture content, water-holding capacity, firmness, stable color, and a consistently lower pH during storage. These findings indicate that plasma-sonic treatment may represent a viable alternative to NaOCl, offering comprehensive preservation of the physicochemical quality of kale during storage.

## 4. Conclusions

In the present study, a non-thermal and non-chlorine decontamination method, referred to as plasma-sonic treatment, was developed by combining PAW and US treatment applied to kale. The effects of plasma-sonic treatment on indigenous bacteria reduction, physicochemical properties, and quality maintenance of kale were comprehensively analyzed. The results demonstrated that plasma-sonic treatment effectively inactivated indigenous bacteria during storage, with efficacy comparable to NaOCl and higher than that of the individual PAW and US treatments. During storage, plasma-sonic maintained the lowest cell counts compared with PAW, US, and NaOCl. Moreover, plasma-sonic treatment demonstrated higher color retention, moisture content, and firmness during storage compared to other groups. Although moisture content significantly decreased during storage, the higher initial moisture content allowed plasma-sonic–treated kale to maintain relatively higher firmness and moisture content compared with NT and NaOCl. Nevertheless, this study did not measure enzymatic activities such as PPO, which are closely associated with chlorophyll degradation. Future research should quantify PPO and other browning-related enzymes to provide biochemical evidence for the color-stabilizing effect of plasma-sonic treatment. Plasma-sonic treatment demonstrates practical scalability because it operates without relying on complex system parameters and instead depends only on several simple and easily controllable variables, such as power and treatment time for PAW and treatment time for US. By adjusting these parameters, the combined plasma-sonic system can maintain consistent reactive species activity and energy delivery across various processing capacities. In addition, this technology can be directly integrated into existing factory facilities or washing lines without modification of the plant layout, making it highly applicable to industrial postharvest processing with minimal installation cost. Furthermore, the elimination of chemical reagents and the reduction of wastewater treatment expenses further enhance its economic efficiency at the industrial scale. However, for practical industrial implementation, several regulatory considerations must be addressed. In the case of using PAW, it is necessary to establish safety standards for residual reactive species, verify microbial control efficacy equivalent to chlorine-based sanitizers, and define hazard analysis and critical control point (HACCP)-based critical limits for key process parameters. For US, regulatory attention focuses on limits of energy exposure, certification of food-contact equipment, and temperature management to prevent product damage. Taken together, plasma-sonic treatment is a promising alternative to NaOCl washing that simultaneously ensures microbial safety and product quality and shows strong potential for practical implementation in fresh-produce processing lines.

## Figures and Tables

**Table 1:** Effects of various treatment (NT, DW, NaOCl, PAW, US, Plasma-sonic) on the inactivation of indigenous bacteria in kale.

Treatment	Viable Cell Count ^2)^ (log CFU/g)	Log Reduction (log CFU/g)	Viable Cell Counts in Rinse Water (log CFU/g)
NT ^1)^	7.2 ± 0.1 ^a^	-	-
DW	5.4 ± 0.1 ^b^	1.8 ± 0.1 ^d^	5.1 ± 0.4 ^a^
NaOCl	4.2 ± 0.3 ^d^	3.0 ± 0.3 ^b^	3.0 ± 0.3 ^c^
PAW	5.3 ± 0.0 ^b^	2.0 ± 0.0 ^d^	3.2 ± 0.1 ^bc^
US	4.9 ± 0.0 ^c^	2.3 ± 0.0 ^c^	4.8 ± 0.1 ^a^
Plasma-sonic	4.0 ± 0.1 ^e^	3.2 ± 0.1 ^a^	3.6 ± 0.2 ^b^

^1)^ NT: unwashed kale; DW: kale washed using DW; NaOCl: kale washed using NaOCl; PAW: kale washed using PAW; US: kale washed using US; Plasma-sonic: kale washed using plasma-sonic. ^2)^ Viable cell counts of unwashed kale: 7.17 ± 0.11 log CFU/g. Mean values followed by different letters significantly differ among viable cell count (*p* < 0.05) according to Tukey’s test with one-way ANOVA.

**Table 2:** Changes in the color and greenness index of kale subjected to various treatments (NT, DW, NaOCl, Plasma-sonic).

Treatment	Color				Greenness Index			
	L* ^2)^	a*	b*	Δ*E*	OD_665_	OD_649_	Chlorophyll a	Chlorophyll b
NT ^1)^	38.52 ± 1.26 ^a 3)^	−10.16 ± 0.99 ^d^	13.02 ± 1.89 ^a^	2.25 ± 1.03 ^b^	0.39 ± 0.03 ^c^	0.34± 0.03 ^a^	4.07 ± 0.12 ^d^	5.95 ± 0.10 ^a^
DW	36.38 ± 0.11 ^bc^	−7.80 ± 0.71 ^b^	7.49 ± 1.05 ^c^	6.40 ± 1.19 ^a^	0.42 ± 0.01 ^c^	0.23± 0.01 ^c^	4.74 ± 0.09 ^c^	3.29 ± 0.08 ^c^
NaOCl	35.78 ± 0.92 ^c^	−6.86 ± 0.07 ^a^	7.44 ± 0.65 ^c^	7.13 ± 0.13 ^a^	0.39 ± 0.00 ^c^	0.20 ± 0.00 ^d^	4.43 ± 0.10 ^cd^	2.75 ± 0.01 ^d^
PAW	37.29 ± 0.30 ^b^	−8.72 ± 0.19 ^c^	10.76 ± 0.25 ^b^	2.98 ± 0.19 ^b^	0.42 ± 0.01 ^c^	0.25 ± 0.01 ^bc^	4.69 ± 0.07 ^c^	3.75 ± 0.09 ^c^
US	36.81 ± 0.21 ^bc^	−11.07 ± 0.48 ^e^	13.35 ± 0.92 ^a^	2.18 ± 0.45 ^b^	0.61 ± 0.01 ^b^	0.33 ± 0.01 ^ab^	6.89 ± 0.11 ^b^	4.69 ± 0.10 ^b^
Plasma-sonic	38.16 ± 0.30 ^a^	−12.24 ± 0.24 ^f^	13.69 ± 0.51 ^a^	2.27 ± 0.37 ^b^	0.74 ± 0.01 ^a^	0.43± 0.01 ^a^	8.28 ± 0.13 ^a^	6.37 ± 0.12 ^a^

^1)^ NT: unwashed kale; DW: kale washed using DW; NaOCl: kale washed using NaOCl; Plasma-sonic: kale washed using plasma-sonic. ^2)^ L*: lightness, a*: redness, b*: yellowness, OD665: optical density at 665, OD649: optical density at 649. ^3)^ Mean values followed by different letters significantly differ among viable cell count (*p* < 0.05) according to Tukey’s test with one-way ANOVA.

**Table 3:** Moisture properties of kale subjected to various treatments (NT, DW, NaOCl, Plasma-sonic).

Treatment	Wet Weight (g) ^2)^	Dry Weight (g)	Moisture Content (%)	Dry Residue (%)	A (%) ^2)^
NT ^1)^	2.0 ± 0.0 ^NS 3)^	0.3 ± 0.0 ^NS^	85.6 ± 0.4 ^bc 4)^	14.4 ± 0.4 ^ab^	694.5 ± 20.8 ^bc^
DW	2.0 ± 0.0 ^NS^	0.3 ± 0.0 ^NS^	85.2 ± 0.7 ^bc^	14.8 ± 0.7 ^ab^	678.6 ± 30.7 ^bc^
NaOCl	2.0 ± 0.0 ^NS^	0.2 ± 0.0 ^NS^	87.3 ± 0.8 ^ab^	12.7 ± 0.8 ^bc^	789.2 ± 50.4 ^ab^
Plasma-sonic	2.0 ± 0.0 ^NS^	0.2 ± 0.0 ^NS^	89.4 ± 0.3 ^a^	10.6 ± 0.3 ^c^	945.9 ± 30.2 ^a^

^1)^ NT: unwashed kale; DW: kale washed using DW; NaOCl: kale washed using NaOCl; US: kale washed using US; Plasma-sonic: kale washed using plasma-sonic. ^2)^ A: Ratio between initial weight and final weight expressed in %. ^3) NS^: No significant. ^4)^ Data are presented as the mean and standard deviation (*n* = 3). Mean values followed by the same letter within a column were not significantly different (*p* < 0.05) among the treatments.

**Table 4:** Changes in the quality attributes of kale subjected to various treatments during storage at 4 °C for 7 days.

Treatment	Storage (d)	Firmness (N)	Color				pH
			L* ^2)^	a*	b*	Δ*E*	
NT ^1)^	0	744.20 ± 158.40 ^c^	40.51 ± 1.17 ^a^	−12.50 ± 1.27 ^b^	7.53 ± 0.02 ^a 3)^	2.54 ± 1.51 ^c^	4.11 ± 0.24
	1	645.68 ± 160.85 ^c^	39.70 ± 1.74 ^NS 4)^	−12.29 ± 1.77 ^NS^	6.37 ± 0.01 ^b^	3.62 ± 1.62 ^NS^	3.17 ± 0.44
	3	610.60 ± 135.71 ^c^	41.60 ± 1.57 ^NS^	−14.26 ± 1.07 ^b^	6.24 ± 0.01 ^a^	4.08 ± 1.99 ^NS^	3.31 ± 0.17
	7	623.54 ± 134.41 ^c^	36.88 ± 0.82 ^NS^	−7.85 ± 0.56 ^a^	6.60 ± 0.04 ^b^	9.71 ± 1.01 ^NS^	3.76 ± 0.41
NaOCl	0	1589.65 ± 200.23 ^b^	34.70 ± 0.51 ^b^	−7.30 ± 0.52 ^a^	6.07 ± 0.01 ^c^	10.94 ± 0.63 ^a^	1.36 ± 1.10
	1	973.69 ± 174.73 ^b^	38.93 ± 2.32 ^NS^	−12.61 ± 1.38 ^NS^	6.49 ± 0.02 ^a^	3.34 ± 2.10 ^NS^	2.96 ± 1.43
	3	722.63 ± 116.43 ^b^	35.35 ± 6.98 ^NS^	−9.51 ± 1.14 ^a^	6.16 ± 0.00 ^b^	8.10 ± 6.73 ^NS^	5.69 ± 0.19
	7	682.79 ± 169.04 ^b^	41.78 ± 1.57 ^NS^	−14.43 ± 0.88 ^c^	6.70 ± 0.00 ^a^	5.40 ± 2.27 ^NS^	5.84 ± 0.07
Plasma-sonic	0	3357.85 ± 713.48 ^a^	36.45 ± 1.35 ^b^	−10.40 ± 0.89 ^b^	6.13 ± 0.00 ^b^	6.18 ± 1.24 ^b^	3.85 ± 0.51
	1	1753.80 ± 289.10 ^a^	35.67 ± 1.79 ^NS^	−11.40 ± 0.73 ^NS^	6.00 ± 0.01 ^c^	5.57 ± 1.69 ^NS^	3.31 ± 0.12
	3	810.62 ± 138.65 ^a^	34.77 ± 1.57 ^NS^	−11.42 ± 1.07 ^a^	6.08 ± 0.02 ^c^	6.63 ± 1.99 ^NS^	3.27 ± 0.21
	7	716.05 ± 117.67 ^a^	36.44 ± 5.16 ^NS^	−11.65 ± 1.34 ^b^	6.33 ± 0.01 ^c^	5.55 ± 5.07 ^NS^	2.65 ± 0.40

^1)^ NT: unwashed kale; NaOCl: kale washed using NaOCl; Plasma-sonic: kale washed using plasma-sonic. ^2)^ L*: lightness, a*: redness, b*: yellowness. ^3)^ Data are presented as the mean and standard deviation (*n* = 3). Mean values followed by the same letter within a column were not significantly different (*p* < 0.05) among the treatments. ^4) NS^: not significant.

## Data Availability

The original contributions presented in the study are included in the article, further inquiries can be directed to the corresponding author.
